# The Hybrid POCUS-to-Echo Feasibility Study: Provision of Expedited Cardiac Point of Care Ultrasound Service (e-POCUS) by the Echocardiography Lab

**DOI:** 10.24908/pocus.v5i1.14224

**Published:** 2020-07-06

**Authors:** Hanane Benbarkat, Tony Sanfilippo, Jian Zhang, Amer M Johri

**Affiliations:** 1 Division of Cardiology, Department of Medicine, Queen's University Kingston, ON Canada; 2 Echocardiography Lab, Kingston General Hospital Kingston, ON Canada

**Keywords:** POCUS, echocardiography

## Abstract

**Background: **Comprehensive transthoracic echocardiography (CTE) provides information vital to the care of acutely ill and unstable patients, but may not be readily available. Cardiac point of care ultrasound (POCUS) is well suited to providing key information at the bedside to expedite decision making. Our objective was to evaluate the feasibility of expedited-POCUS (e-POCUS) provided by the echo lab for internal medicine, cardiology and intensive care services. **Methods: **A new e-POCUS service was developed by the Kingston Health Sciences Center Echo Lab, whereby focused information relevant to 4 clinical situations (acute heart failure, tamponade, shock and suspected acute valvulopathy) would be provided urgently at the bedside. Requests were acquired over a 4 month period. Sonographers were immediately deployed on request and followed a standard POCUS protocol for each scenario. Staff echocardiographers provided immediate interpretation and arranged for further imaging at their discretion. The response time, diagnostic accuracy and clinical utility of e-POCUS was assessed. **Results: **A total of 18 patients were evaluated. The average time of an e-POCUS exam was 10 minutes and the average e-POCUS to formal CTE timing was 1.3 days. The agreement between e-POCUS and CTE for the presence of segmental wall motion abnormalities was 83% (Kappa=0.61, p=0.009) and 72% for the detection of right ventricular dilatation (Kappa =0.44, p=0.058). The e-POCUS results altered the working diagnosis in 72% of cases. **Conclusion: **The provision of an e-POCUS service by the Echo Lab is a feasible workflow solution meeting the demands of a new practice pattern.

## Background

Echocardiography is not consistently available to acutely ill patients. The barriers include scheduling, availability of a limited number of full-service units, and the need to follow strict protocol driven procedures to ensure a full-standardized study. These barriers have recently resulted in the proliferation of cardiac point of care ultrasound devices (POCUS) to allow for decision-making immediately at the bedside. However, concerns about the quality of cardiac POCUS acquired by non-traditional operators remains, in addition to concerns related to interpretation and archiving 1. To address these concerns while simultaneously meeting the demand for immediate decision-making, expedited POCUS (e-POCUS) sonographers in an echocardiography lab may be a solution to providing high quality imaging that is rapidly delivered at the bedside. In our study, we identified four clinical scenarios where e-POCUS would be useful: 1) suspicion of acute heart failure, 2) tamponade, 3) shock of unknown aetiology and 4) Clinical suspicion of a significant valvulopathy. Our objective was to evaluate the feasibility of e-POCUS provided by the Echo Lab for the cardiology, internal medicine and intensive care services.

## Methods

Education of the sonographers and referring physicians: Six experienced sonographers were given thirty-minute hands on session on the use of the e-POCUS machine (Mindray TE 7)^2^ and on how to transfer the images to a digital storage and reporting system (Philips Healthcare). The Mindray TE 7 machine has a 15-inch anti-glare touch screen with image optimization options such as zoom and gain adjustments, which can be selected with a single touch.

No electrocardiogram is required during image acquisition. For the comprehensive transthoracic echocardiogram (CTE), a Vivid E9 (GE Healthcare) was used. The cardiology, internal medicine and intensive care unit (ICU) department heads and chief residents were informed that the Echo Lab was providing an e-POCUS service in the evaluation of these four clinical scenarios.

### Clinical Care Pathway (Figure 1)

The e-POCUS service was available from Monday to Friday, 8:00 to 16:00. When a call was made to the e-POCUS service, the assigned sonographer would immediately proceed to the bedside to answer the immediate question following a pre-specified protocol for each clinical situation. The images were then transferred to the cardiologist reader of the day who would make an immediate interpretation following the American Society of Echocardiography (ASE) guidelines 3. The interpretation of valvular lesions from the e-POCUS scan was based on qualitative visual assessment and was only reported as significant if it appeared moderate to severe visually. The results were then communicated to the referring service by phone and/or pager. The referring physician was also asked whether the e-POCUS confirmed or changed the pre-POCUS working diagnosis, which was always documented before the e-POCUS protocol was initiated. If no initial abnormality was found on the ‘scout’ exam, the study was triaged as per normal routine protocol. If an initial concerning abnormality was found, treatment decisions were at the discretion of the referring physician, and a full-service echocardiogram was urgently performed to supplement the ‘scout’ images. The longest delay allowed between the e-POCUS and CTE was 48 hours. Both the e-POCUS scout images and CTE study were archived and reported individually in the hospital electronic medical record. The time to respond and to acquire the e-POCUS and CTE were recorded. 

**Figure 1:**
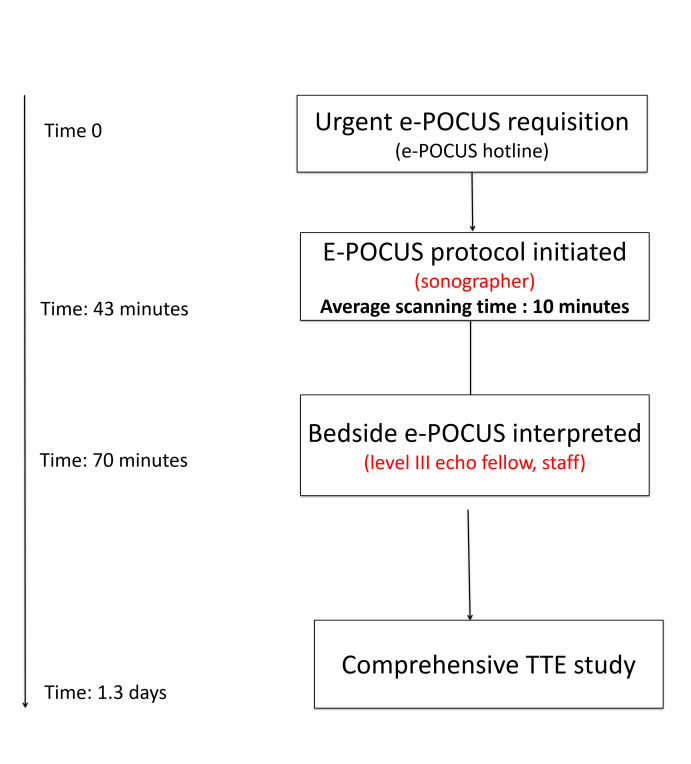
Flowchart of the e-POCUS service with the average timing of each step starting from the time of the requisition (time 0).

### Statistical analysis

The continuous data are expressed as mean ± one standard deviation and categorical variables as number (%) of the total group. Results from the CTE were considered the gold standard. The agreement between e-POCUS and CTE was assessed using the Kappa statistics. Comparisons between continuous variables were done using Student t test.

## Results

A total of 18 cases were collected over a 4-month period. Fifteen cases were requested from the internal medicine department, two cases from the ICU and one case from the cardiology department. Diagnostic imaging was collected in 17 cases. There was one patient where imaging was not diagnostic because of difficult positioning and clinical instability with the use of non-invasive ventilation (BiPAP). Most of the requests were for acute heart failure (50%) followed by shock (22%), tamponade (17%) and acute valvulopathy (11%). There were four calls made to the e-POCUS service that were not appropriate. Three of these requests were made to rule out vegetation in patients without any physical findings of acute valvulopathy. The other case was a patient who had elevated troponins and the requesting service wanted to rule out any regional wall motion abnormality.

**Figure 2:**
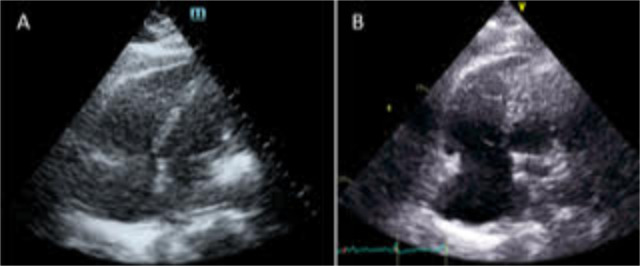
A) E-POCUS, B) Comprehensive transthoracic echocardiogram (CTE) A case of a 75-year-old woman presenting to ER with acute shortness of breath and desaturation. E-POCUS and CTE revealed significant RV dilatation with severe systolic dysfunction.

The average scan duration was 10 minutes. The average of stored images was 26 ± 8 and 82 ± 26 for the e-POCUS and CTE respectively. The difference in the number of stored images between e-POCUS and CTE was statistically significant (p <0.0001). The time from the request to the start of the POCUS exam was 43 minutes. The results of the POCUS were communicated to the treating team within 70 minutes of the request. The CTE was done within 1.3 days. There was one patient that was inadvertently discharged from hospital before having his CTE. However, he was called back for it within 7 days of the e-POCUS. No major finding was missed in this patient. The agreement between e-POCUS and CTE for the presence of regional wall motion abnormalities (RWMA) was 83% (Kappa=0.61, p=0.009) and 72% for the detection of right ventricular dilatation (Kappa =0.44, p=0.058). In one case, the grading of tricuspid regurgitation was upgraded from mild to moderate. Overall, no major finding was missed. The e-POCUS altered the working diagnosis of the referring physicians in 72% of cases (13/18 cases). For example, one of the cases was a 75 year-old woman presenting to emergency room (ER) with acute shortness of breath and desaturation (Figure 2). The CXR showed interstitial lung markings. The working diagnosis was acute heart failure. She was treated with intravenous furosemide in the ER without any improvement. E-POCUS was requested by the internal medicine in the following 24 hours. The main findings were significant right ventricular dilatation with severe systolic dysfunction. The left ventricular function was normal. Consequently, the treating team stopped diuretics and started investigations for pulmonary hypertension.

## Discussion

Our study was designed to assess the feasibility of providing an expedited POCUS (e-POCUS) evaluation by experienced sonographers to the cardiology, internal medicine and ICU departments in a busy tertiary care Echo Lab. We found that it was easily feasible and useful because the working diagnosis of the referring physicians was altered by e-POCUS exam in 72% of the cases.

E-POCUS diagnostic limitation: The fact that e-POCUS missed subtle RWMA is in keeping with previous work. Other studies have shown that RWMA with POCUS are more likely to be missed compared to the CTE 4. On the other hand, POCUS and CTE have a good correlation for LV function assessment 4. The Kappa and percent of agreement for the presence of RWMA in our study was good and it was statistically significant (Kappa=0.61, p=0.009). The cases where RWMA was missed were all identified on the CTE where in one case Definity contrast had to be used for better evaluation. 

The detection of RV dilatation was more in the moderate inter-rater agreement range and the p-value was just short of significance due the small sample size (Kappa =0.44, p=0.058). In fact, it is sometimes challenging to assess the RV size due to the limited views of the RV that are obtained. In our study, the cases where there was disagreement were mostly patients where RV dilatation was subsequently seen on CTE where more views of the RV are usually obtained. On the other hand, the assessment of RV systolic function was the same with e- POCUS (visual estimate) and CTE (visual estimate supported by more objective measurements). Lastly, the case where mild tricuspid regurgitation was upgraded to moderate is in keeping with the limitations of POCUS to grade valvular lesion severity. However, it has been shown that there is a good agreement between POCUS and the CTE for the detection of significant valvulopathy 5. 

### Study limitations

Our study is a single institution project that included a small number of patients. The interpreter of the CTE was not blinded to the POCUS results, which may affect their interpretation. The interpretation of the e-POCUS and CTE were completed by the same reader in 55% of the cases (10 out of 18 cases), which may introduce an element of bias. On the other hand, this approach reflects the day-to-day practice where the echocardiographers rotate through the Echo Lab and don’t have control over which case they can read.

## Conclusion

The provision of an expedited cardiac POCUS (e-POCUS) service by the echocardiography lab is feasible and potentially a rapid workflow solution meeting the demands of care providers. The new workflow model offers a potentially new organizational approach to the Echo Lab of the near future, providing rapid imaging, education, and guidance to various hospital departments in a “hub-and-spoke”, centered around a high quality, accredited Unit.

## Conflicts of Interest

None declared.
